# Identification of a novel competing endogenous RNA network and candidate drugs associated with ferroptosis in aldosterone-producing adenomas

**DOI:** 10.18632/aging.205028

**Published:** 2023-09-13

**Authors:** Yu Hanxiao, Yang Boyun, Jia Minyue, Song Xiaoxiao

**Affiliations:** 1 Clinical Research Center, The Second Affiliated Hospital, Zhejiang University School of Medicine, Hangzhou, China; 2 Department of Allergy, The Second Affiliated Hospital, Zhejiang University School of Medicine, Hangzhou, China; 3 Department of Ultrasound, The Second Affiliated Hospital, Zhejiang University School of Medicine, Hangzhou, China; 4 Department of Endocrinology and Metabolism, The Second Affiliated Hospital, Zhejiang University School of Medicine, Hangzhou, China

**Keywords:** aldosterone-producing adenomas, ferroptosis, competing endogenous RNA, candidate drugs, differentially expressed genes

## Abstract

Aldosterone-producing adenoma (APA), characterized by unilaterally excessive aldosterone production, is a common cause of primary aldosteronism. Ferroptosis, a recently raised iron-dependent mode of programmed cell death, has been involved in the development and therapy of various diseases. This study obtained datasets of the mRNA and lncRNA expression profiles for APA and adjacent adrenal gland (AAG) from the Gene Expression Omnibus (GEO) database. Differentially expressed genes (DEGs) and lncRNAs (DE lncRNAs) associated with ferroptosis were identified. Enrichment analyses indicated 89 ferroptosis-related DEGs were primarily enriched in ROS related processes and ferroptosis. Two physical cores, and one combined core were identified in the protein–protein interaction (PPI). DEGs and clinical traits were used in conjunction to screen eight hub genes from two hub modules and 89 DEGs. A competitive endogenous RNA (ceRNA) network was constructed via co-express analysis. Thereafter, molecular docking was used to identify potential targets. Two active compounds, QL-X-138 and MK-1775, bound to AURKA and DUOX1, respectively, with the lowest binding energies. Molecular dynamics simulation verified the stability of the two complexes. In summary, our studies identified eight hub genes and a novel ceRNA regulatory network associated with ferroptosis, wherein QL-X-138 and MK-1775 were considered to be potential drugs for treating APA.

## INTRODUCTION

Primary aldosteronism caused by increased aldosterone levels is well established to be linked to secondary hypertension, with an estimated prevalence of nearly 6% in 1.1 billion hypertensive patients of the worldwide [1, 2], and a prevalence of up to 20% in resistant hypertensive patients [3]. The diagnosis of primary aldosteronism is based on hypertension related to an elevated aldosterone-to-renin ratio and usually hypokalemia [4, 5]. APA as well as idiopathic hyperaldosteronism [4] are the major causes of primary aldosteronism, which is characterized by autonomous production of aldosterone in the adrenal cortex [6]. Although the renin-angiotensin-II-aldosterone system has vigorous feedback regulation, aldosterone production in APA is normally uncontrollable even under low renin conditions [7]. Somatic mutations are known to promote the production of aldosterone, along with the associated genes that mainly encoding ion channels such as KCNJ5 [8], CACNA1D [9, 10], CACNA1H [11], CLCN2 [5, 12] and ATPases (ATP1A1 [9] and ATP2B3 [13]) result in elevating Ca^2+^ concentration in zona glomerulosa cells [14, 15]. Although most APAs can be correctable for hypertension via adrenalectomy, only 37% of patients achieve complete clinical success [16]. Thus, noninvasive treatment, fewer side effects and improved drug tolerance are necessary to either compete for the mineralocorticoid receptor with aldosterone or suppress the Ca^2+^ concentration [17]. Novel target drugs can be identified by programmed cell death of tumor cells and regulatory networks combined with the aldosterone production pathway through gene expression profile.

Programmed cell death can be classified into six forms: apoptosis, necroptosis, autophagy, ferroptosis, pyroptosis and necrosis [18]. Since the discovery of the iron-dependent form of cell death termed ferroptosis [19], there has been a rapid increase in research on the same [20]. Ferroptosis is different from other programmed cell deaths in terms of morphology, biochemical features and regulatory genes [19, 21]. It depends on the accumulation of cell ROS and excess lipid peroxidation, which induce oxidative damage in the cell membrane [19]. Excessive iron causes lipid peroxidation through ROS production and the iron-associated Fenton reaction [22]. Moreover, certain proteins that transfer membrane electrons, such as NADPH oxidases (NOX) [19, 23], also have a positive effect on lipid peroxidation via ROS production in tumors [24]. In contrast, growing evidence has approved that some drugs, including ROS-inducing drugs [25, 26], cytokines [27] and ionizing radiation [28], can function as proferroptotic inducers. Conversely, antiferroptotic inhibitors can cause inflammation-related immune suppression [24]. Ferroptosis may be inhibited in malignant tumors [29] and activated in neurodegenerative diseases [30]. Recently Yuhong et al. revealed that the differentially expressed gene BEX1 had a potent effect on protecting adrenocortical cells from ferroptosis in micro-APAs compared to macro-APAs [15]. The sequencing on APA with or without KCNJ5 mutation of spatial metabolomics showed abundant accumulation of proferroptotic metabolites, such as adrenic acid and docosapentaenoic acid [31]. lncRNAs also engage in the ferroptosis process by impacting post-transcriptional alterations and protein stability, as observed in contemporary studies [32]. However, the molecular mechanisms by which ferroptosis-related genes and lncRNAs regulate aldosterone production remain unclear.

Fortunately, with the improvement of sequencing and bioinformatics, large sample sizes data from multiple independent studies can be integrated and explored potential information by researchers. Yang et al. reported the relationship between tumor immune genes and prognosis in ovarian cancer [33] and breast cancer [34, 35] based on the multiple cohorts in the TCGA and GEO databases. In this study, we download mRNA and lncRNA expression profile of APA and AAG tissue with corresponding clinical characteristics from the GEO database. Subsequently, the ferroptosis-related DEGs and DE lncRNAs were screened out, and the enriched pathway was analyzed by Metascape and Gene Set Enrichment Analysis (GSEA). Based on the ferroptosis-related DEGs and DE lncRNAs, a ceRNA network was constructed by predicting target miRNAs. Finally, drugs targeting hub genes related to ferroptosis were anticipated by molecular docking and molecular dynamics simulation.

## RESULTS

### Identification of DE lncRNAs and ferroptosis-related DEGs

**Figure 1:**
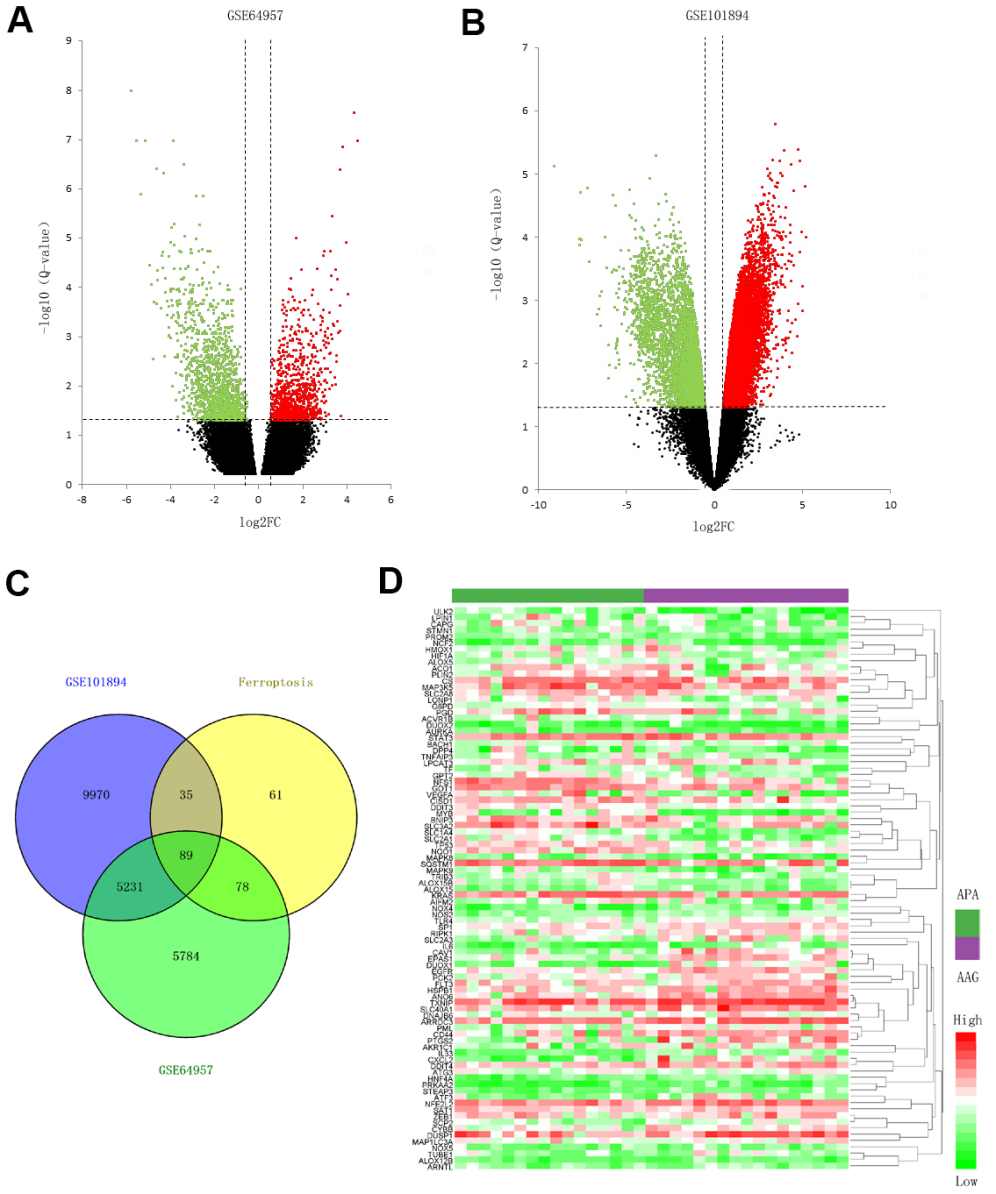
**Screening for the ferroptosis-related DEGs.** (**A**, **B**) Volcano plots of the DEGs in GSE64957 and GSE101894 between APA and AAG tissue. The red dots represent the upregulated genes, the green dots represent the downregulated genes, and the black dots represent genes with no significant difference. (**C**) Venn diagram to identify ferroptosis-related DEGs. (**D**) Expression heatmap of 89 ferroptosis-related DEGs in APA and AAG tissue.

The datasets of GSE64957 and GSE101894 were selected to identify the DEGs and DE lncRNAs in APA tissue compared to that in the AAG tissue, as depicted in the volcano plots (Figure 1A, 1B). In addition, 263 ferroptosis-related genes from FerrDb were used to screen the ferroptosis-related DEGs. Consequently 89 ferroptosis-related DEGs were identified, including 44 upregulated genes and 45 downregulated genes (Figure 1C). The expression of 89 genes in the APA and AAG tissue are presented in the heatmap (Figure 1D).

### Enrichment analysis of 89 ferroptosis-related DEGs

**Figure 2:**
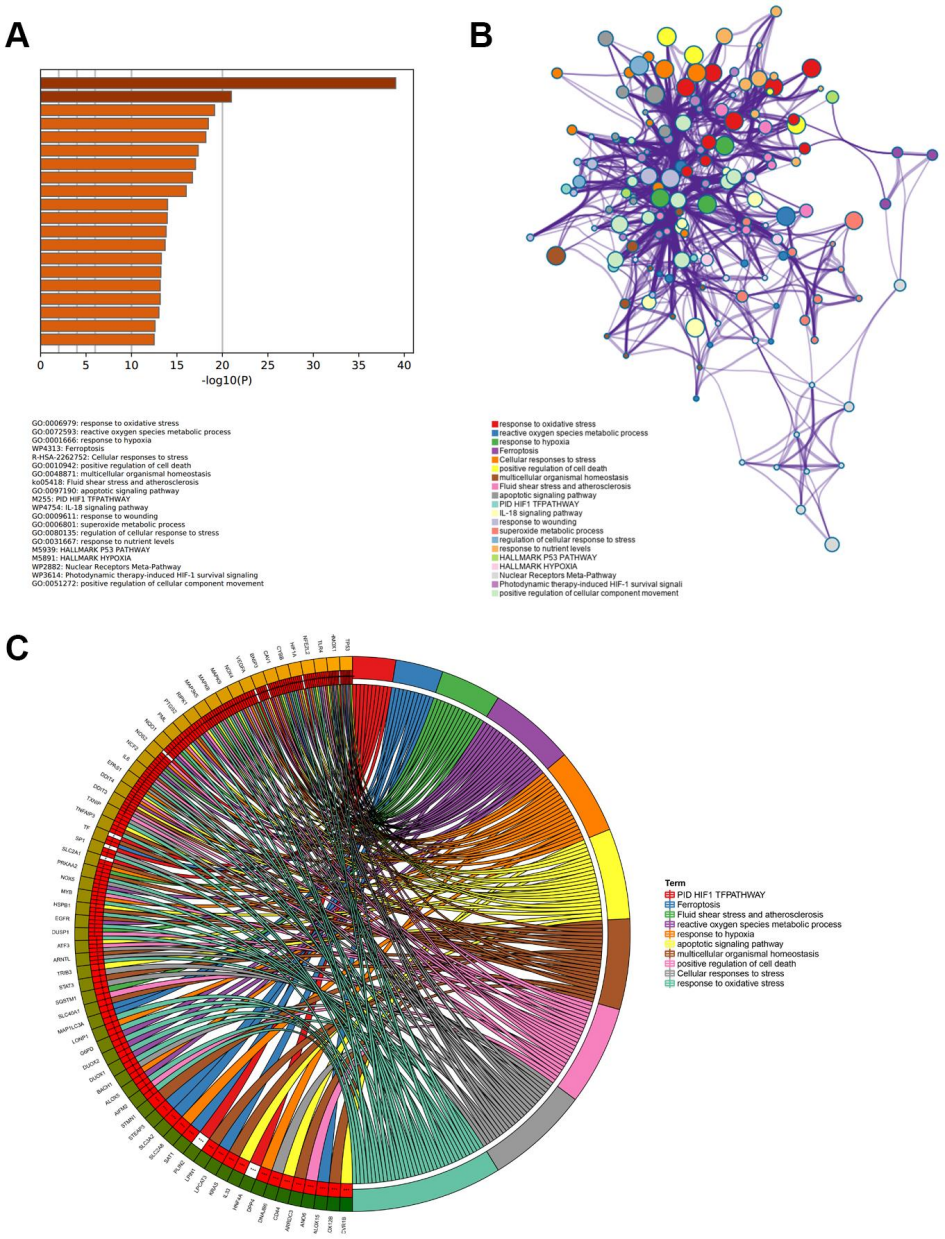
**Enriched functions and pathways of ferroptosis-related DEGs.** (**A**) The top 20 most significant enriched terms analyzed from Metascape, ranked according to –log_10_ (P) value. (**B**) The interaction network of 20 enriched terms, each identical color circle represents an enriched term. (**C**) The relationship between ferroptosis-related DEGs and top 10 enriched terms.

Metascape was used to analyze the functions and pathways of 89 ferroptosis-related DEGs in patients with APA. This tool could identify all statistically enriched terms that were selected in this study, including Gene Ontology biological processes (GO BP), reactome gene sets, KEGG pathways, canonical pathways, and other pathways. Then, the enrichment factors and accumulative hypergeometric *P*-values were calculated for the filtrate. Based on the Kappa-statistical similarities among the enriched genes, the remaining significant terms were hierarchically clustered into a tree [36]. The top 20 most highly enriched items are presented in Figure 2A. Among these items, the response to oxidative stress, reactive oxygen species metabolic process, superoxide metabolic process and positive cell death regulation of were associated with the production of oxidative stress in the GO BP category. This suggested that these genes enriched in the items played a role in the homeostasis and metabolism of superoxides. Furthermore, KEGG pathway demonstrated that the DEGs were mainly concentrated in the ferroptosis, hypoxia-inducible factor 1 (HIF1) TF (involved in the response to hypoxia), and IL-18 signaling pathways (Figure 2A). Upon selecting the top 20 terms from the clusters of functions and pathways, this tool transformed these terms into a network exhibition (Figure 2B). When the Kappa score was greater than 0.3, the node network could be connected. More specifically, each term was represented by a circle whose size was proportional to the number of genes enriched in that term. In addition, the same function or pathway clusters were indicated by an identical color. These terms were connected to an edge using a similarity score, which determined the thickness of the edge. The GO BP items, response to oxidative stress and reactive oxygen species metabolic processes, had a greater number of genes. Furthermore, they were connected to the ferroptosis pathway and positive regulation of cell death more intensively. To further investigate the relationship between 89 ferroptosis-related DEGs and enriched function as well as pathways, a chord plot was drawn to visualize the DEGs and top 10 enriched terms (Figure 2C). Their responses to oxidative stress, cellular responses to stress and positive regulation of cell death were shown to comprise the major genes. Twenty-three DEGs enriched more than three terms suggesting that they participated in regulating multiple pathways and played a key role in ferroptosis-related functions.

### PPI network analysis, physical and combined cores

**Figure 3:**
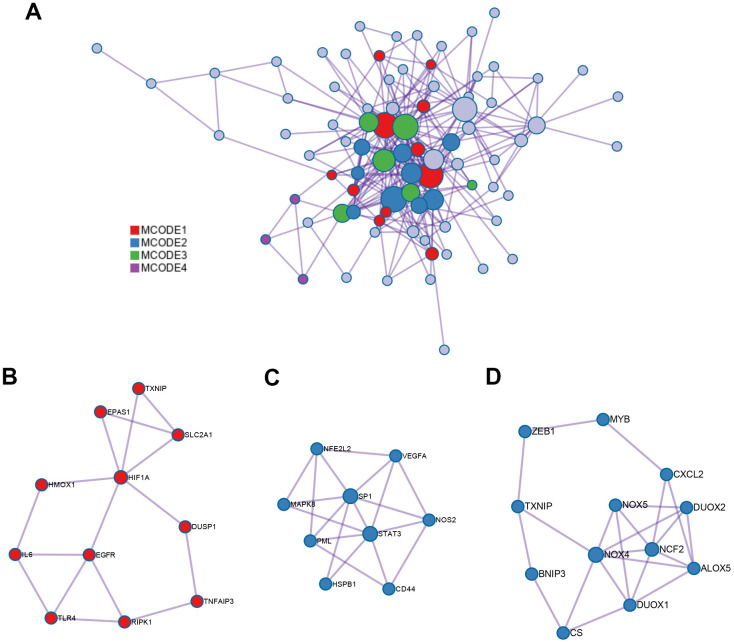
**PPI network and three MCODE clusters.** (**A**) A connected interaction network of 89 ferroptosis-related DEGs. (**B**, **C**) Two physical cores extracted from the PPI network. (**D**) The combined core extracted from the PPI network. Each node represents a protein, while each edge represents the relation of protein-protein.

The interaction network of proteins coded by 89 ferroptosis-related DEGs was constructed by Metascape using the MCODE module (Figure 3A). The MCODE module was utilized to screen gene clusters, as per the algorithm described in this method. Three key subnetworks were identified if the network contained between three and five hundred proteins, and one interacted with at least one other member in the list (Figure 3B–3D). The two physical cores contained 11 (Figure 3B) and 9 DEGs (Figure 3C), respectively, suggesting that these DEGs regulated more physical pathways. Furthermore, functions and pathways enriched analyses were independently applied to the three key subnetworks. The DEGs in the first physical core were primarily associated with the cellular response to chemical stress, response to oxidative stress, as well as cellular response to oxidative stress (Figure 3B). DEGs in the second physical core were strongly related to the AGE/RAGE signaling pathway, which played an important role in the production of ROS mediated by NADPH or derived from NOX (Figure 3C) [37, 38]. The combined core, comprising 12 DEGs, had the greatest number of nodes and edges among all the subnetworks (Figure 3D). It was associated with the apoptotic signaling and extrinsic apoptotic signaling pathways, suggesting the involvement of alternative programmed cell death. Moreover, the NOX family (including NOX4, NOX5, DUOX1, and DUOX2 in this combined core) was demonstrated to contribute both to ferroptosis and apoptosis by generating ROS [39]. This combined core might comprise an axis such as the FBW7-NRA41-SCD1 axis [40] that synchronously regulated ferroptosis and apoptosis.

### WGCNA and hub genes identification

**Figure 4:**
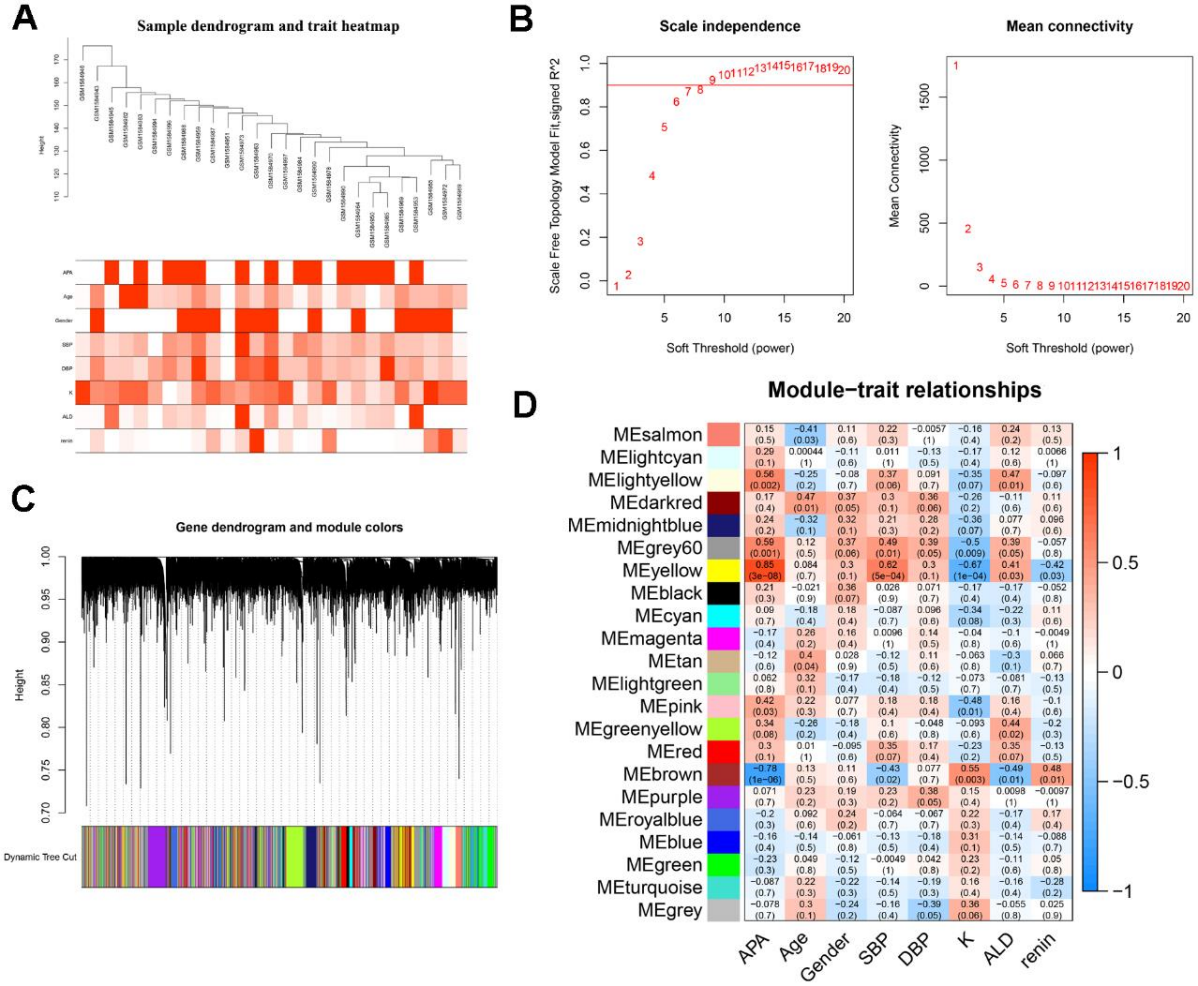
**WGCNA analysis and identification of hub modules related to the clinical traits.** (**A**) Clustering dendrograms of samples and clinical traits of APA, age, gender, SBP, DBP, K, ALD and renin showed at the bottom. (**B**) Analysis of scale free network topology for various soft-threshold powers. The image on the left demonstrates the effect of soft-threshold power on the scale free topology model fit index; the right image shows the effect of soft-threshold power on the mean connectivity of the network. (**C**) Gene clustering dendrograms with dissimilarity based on the topological overlap, assigned with specific module colors. (**D**) The heatmap of correlation coefficient and *p*-value between modules and clinical traits. Each row represents a module, and each column corresponds to a clinical trait.

**Figure 5:**
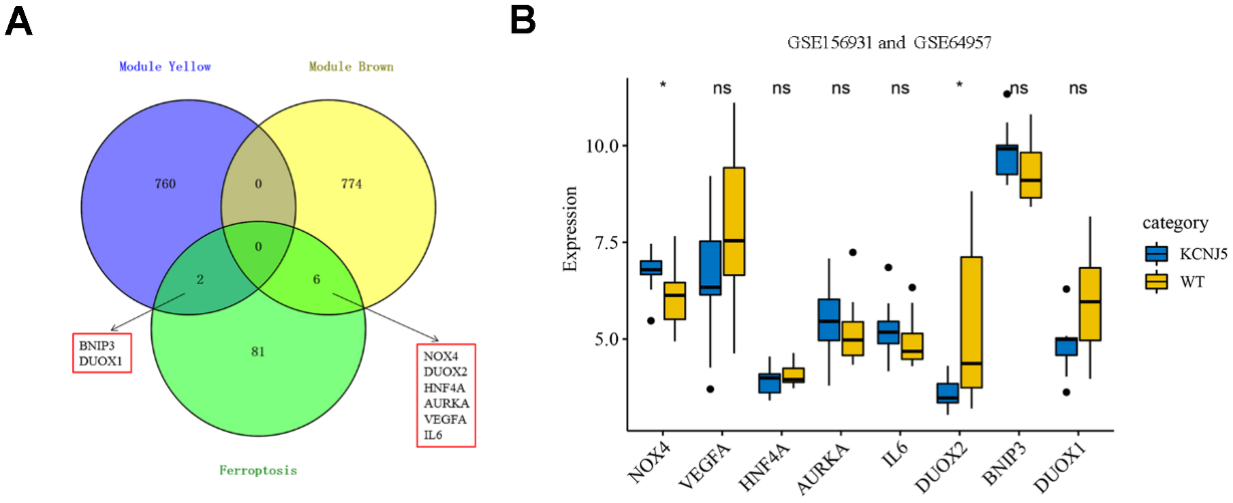
**Identification of eight hub ferroptosis-related DEGs and the expression among mutant KCNJ5 and WT APA samples.** (**A**) Identification of BNIP3, DUOX1, DUOX2, NOX4, HNF4A, AURKA, VEGFA and IL6 from the intersection of hub module yellow and brown, along with 89 ferroptosis-related DEGs. (**B**) The expression of eight hub genes among the mutant KCNJ5 and WT groups. ^*^P < 0.05.

To screen the hub genes from all genes associated with clinical characteristics, the genes expression profile and clinical traits of patients with APA were combined to construct a gene co-expression network. Weighted gene co-expression network analysis (WGCNA) is a bioinformatic analysis that is used to investigate the relationships between genes and phenotypes efficiencies [41]. The clinical traits, including APA, age, gender, systolic blood pressure (SBP), diastolic blood pressure (DBP), serum K^+^ (K), plasma aldosterone (ALD), and plasma renin of APA as well as AAG samples were clustered and depicted on a heatmap (Figure 4A). When the soft threshold was set 8, the index of scale-free topological network was 0.9. Hence, the network was closer to the actual network (Figure 4B). The created gene dendrograms and corresponding module colors were drawn in Figure 4C. The genes were divided into 22 modules obtained from the visualized correlations between module eigengenes and clinical characteristics (Figure 4D). APA has a complex pathogenic mechanism of involving serum K+, plasma aldosterone and renin et al. Here, we took multiple clinicopathological factors into consideration and analyzed the correlation between each gene module. The APA, SBP and ALD of clinical characteristics were more positively relevant to the module yellow, and more negatively relevant to the module brown of all modules. Moreover, the most positive and negative correlation coefficient between APA and module yellow as well as brown were 0.85 and -0.78, respectively. This indicated that the genes in module yellow may contribute to the pathogenesis and development of APA, SBP and ALD. In contrast, the genes in module brown may repress the progression of APA, SBP and ALD. The hub genes were identified from the intersection of modules yellow and brown, along with that of ferroptosis-related DEGs (Figure 5A). Remarkably, IL6 and VEGFA were members of physical cores, whereas NOX4, DUOX1, DUOX2, and BNIP3 interacted with other genes in the combined core from PPI network (Figure 3B–3D). This suggested that these genes played an important role in the subnetworks.

### The expression of eight hub genes in mutant KCNJ5 and WT APA samples

Somatic mutations are a common cause of APA. The general prevalence of KCNJ5 mutations was observed to be 43% in patients with APA [42]. The ComBat was used to remove the batch effect to compare the expression of eight hub genes, and the PCA diagram showed the before and after correction (Supplementary Figure 1). The expression of NOX4 and DUOX2, two members of NOX family, was significantly differentiated in mutant KCNJ5 samples compared to that in the WT (Figure 5B). This indicated the impact of the KCNJ5 mutation on the expression of ferroptosis-related genes.

### Construction of ceRNA network

**Figure 6:**
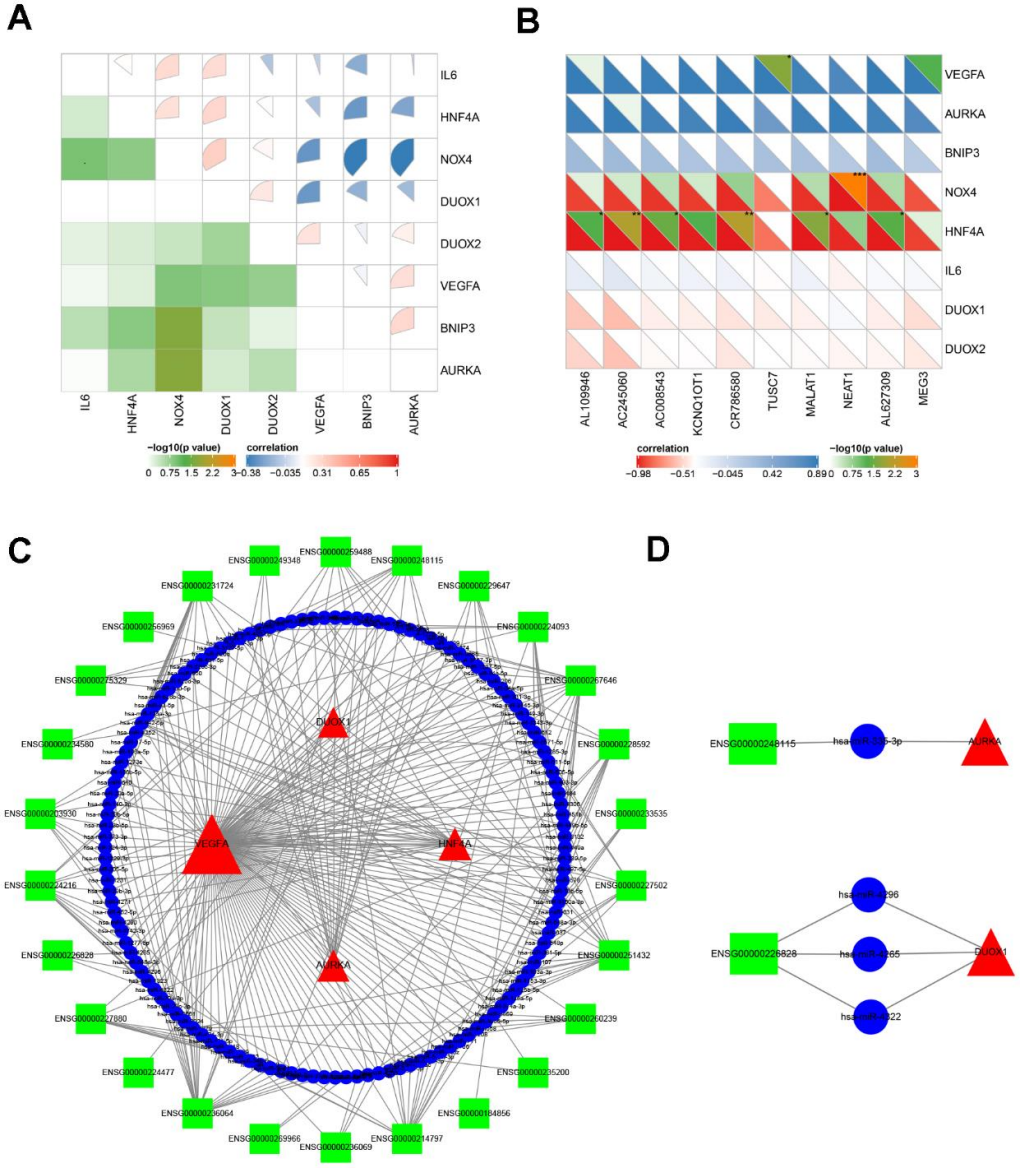
**The analysis of molecular crosstalk.** (**A**) The correlation between eight hub ferroptosis-related DEGs. (**B**) The correlation analysis between 8 hub ferroptosis-related DEGs and 10 DE lncRNAs. (**C**) The ceRNA network interaction among lncRNAs, miRNAs, and mRNAs. (**D**) Two axes ENSG00000248115-hsa-miR-335-AURKA and ENSG00000226828- hsa-miR-4265/hsa-miR-4296/hsa-miR-4322-DUOX1. Green squares represent lncRNAs; blue circles represent miRNAs; red triangles represent mRNAs; the size of the shape represents the degree of connection. ^*^P < 0.05; ^**^P < 0.01; ^***^P < 0.001.

It is well known that miRNAs are recognized as targeted regulators by binding mRNAs. The lncRNA-miRNA-mRNA regulatory network across transcriptional talk is formed as a ceRNA [43]. This study identified miRNAs predicted by online tools using gene-miRNA and miRNA-lncRNA target modules. The miRNAs were selected by the targeted intersection of eight hub ferroptosis-related DEGs and correlated DE lncRNAs. The key lncRNA emerged according to the positively correlated to the expression of mRNAs and that located in the cytoplasm. Consequently, based on the interaction of lncRNA-miRNA-mRNA, the ceRNA was constructed with 26 lncRNAs, 132 miRNAs and 4 mRNAs (Figure 6C). Among all the predicted miRNAs, the ones with the highest degree of connection were miR-942, miR-342, miR-877, miR-511, miR-655, miR-4279, miR-3121, miR-339, miR-5571 and miR-1343. This result identified couples of lncRNA-miRNA-mRNA associations, including lnc-RASL11B (ENSG00000248115)-miR-335-AURKA, and lnc-ZBTB18 (ENSG00000226828)-miR-4296/4265/4322-DUOX1 axes, which might be important therapeutic targets for APA.

### The impact on co-expression of eight hub ferroptosis-related DEGs and DE lncRNAs

To further elucidate the pathogenesis of APA at the transcriptome level, the co-expression of genes and lncRNAs was analyzed. All eight hub ferroptosis-related DEGs correlated with one another at different levels (*P* < 0.05) (Figure 6A). Among them, AURKA and BNIP3, AURKA and VEGFA, VEGFA and DUOX2, DUOX1 and NOX4, DUOX1 and HNF4A, DUOX1 and IL6, NOX4 and HNF4A, NOX4 and IL6 were the eight most positively correlated pairs of genes. In contrast, the gene pairs with the most negative correlations were HNF4A and BNIP3, NOX4 and VEGFA, NOX4 and BNIP3, NOX4 and AURKA, DUOX1 and VEGFA. The strong correlation between the eight genes suggested that the genes may impact one another during their expressions in APA. Moreover, to further understand the expression of these eight genes, which are regulated by DE lncRNAs, we selected the top 10 DE lncRNAs with the highest degree of connection using Pearson’s correlation coefficient (Figure 6B). Consequently, a strong positive correlation was observed between TUSC7 and VEGFA. However, the lncRNAs AL109946, AC245060, AC008543, CR786580, MALAT1, AL627309 and genes HNF4A, NEAT1 and NOX4 exhibited a strong negative correlation.

### GSEA analysis of the top 10 lncRNAs

**Figure 7:**
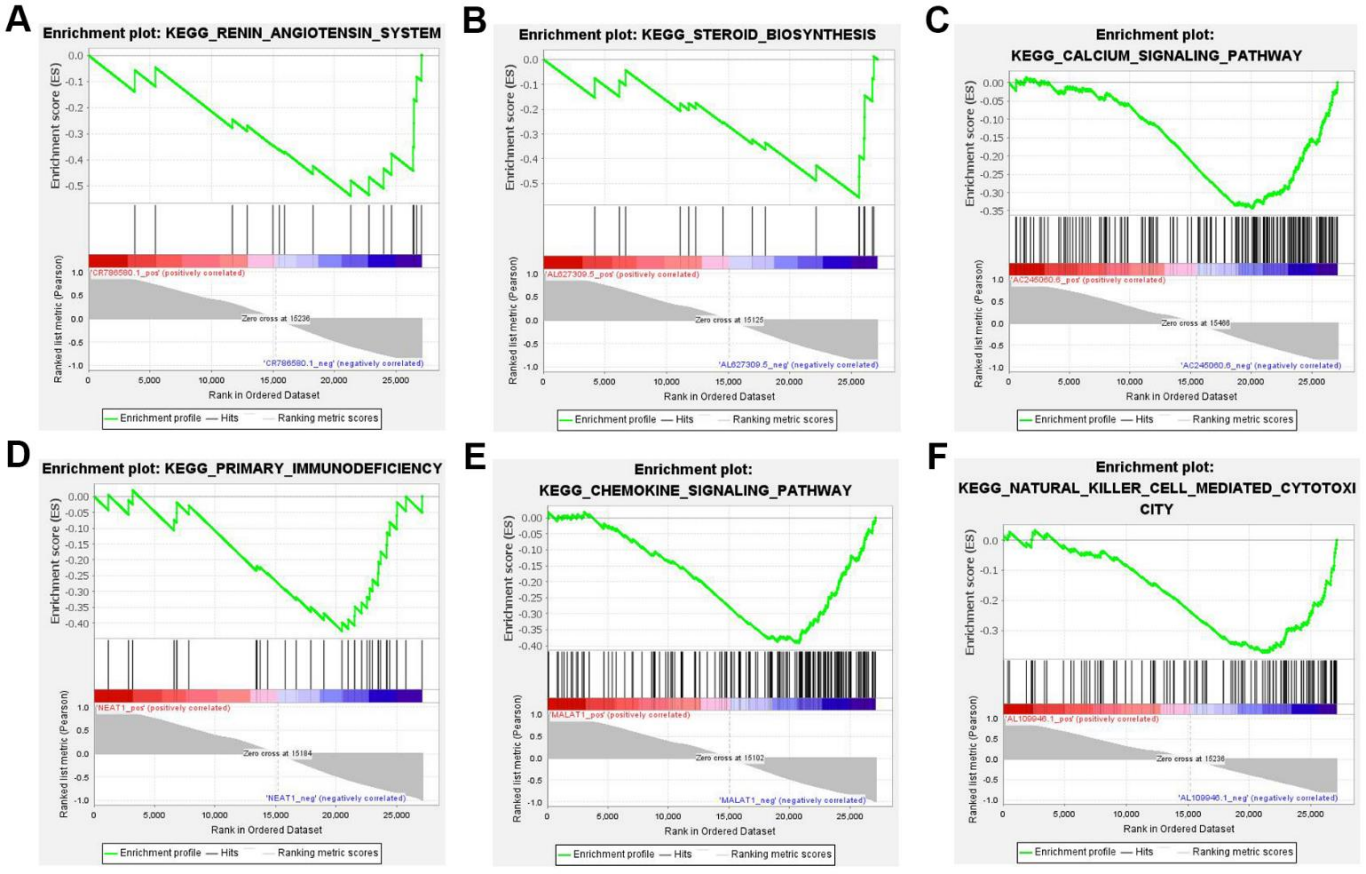
**Six most significant enriched KEGG pathway of 10 lncRNAs.** (**A**) Renin-angiotensin system (NES=-1.913, *P <* 0.05). (**B**) Steroid biosynthesis (NES=-1.999, *P <* 0.05). (**C**) Calcium signaling pathway (NES=-2.162, *P <* 0.05). (**D**) Primary immunodeficiency (NES=-1.884, *P <* 0.05). (**E**) Chemokine signaling pathway (NES=-2.475, *P <* 0.05). (**F**) Natural killer cell mediated cytotoxicity (NES=-2.118, *P <* 0.05). NES: normalized enrichment score.

In contrast to the gene enrichment of functions and pathways, the top 10 DE lncRNAs enriched pathways that showed the highest degree of connection with GSEA were also analyzed. The results revealed the majority of 10 lncRNAs regulated immune and aldosterone-biosynthesis related pathway, such as the renin-angiotensin system, steroid biosynthesis, calcium signaling pathway, primary immunodeficiency, chemokine signaling pathway, and natural killer cell mediated cytotoxicity (Figure 7).

### Prediction of candidate drugs through CMAP

To further enhance the precision of drugs screening, 44 upregulated and 45 downregulated ferroptosis-related genes were queried on the CMAP. The detailed results showed that the top ranked drugs, including fludrocortisone and fluocinolone, were positively associated with APA suggesting that they promoted APA progression or aldosterone production (Table 1). Conversely, the bottom ranked drugs that were negatively associated with APA included MK-1775, GW-843682X, QL-X-138 and OM-137, which were the candidate drugs. Drugs with potent adverse reactions or potential toxicities were excluded from this study.

**Table 1:** The positive and negative small molecular compounds for APA obtained from the connectivity map (CMAP) database.

**Rank**	**CMAP name**	**Belongs to**	**Canonical SMILES**
1	Apicidin	HDAC^1^ inhibitor	CCC(C)C1C(=O)N2CCCCC2C(=O)NC(C(=O)NC(C(=O)N1)CC3=CN(C4=CC=CC=C43)OC)CCCCCC(=O)CC
2	Entinostat	HDAC inhibitor	C1=CC=C(C(=C1)N)NC(=O)C2=CC=C(C=C2)CNC(=O)OCC3=CN=CC=C3
3	Fludrocortisone	Glucocorticoid receptor agonist	CC12CCC(=O)C=C1CCC3C2(C(CC4(C3CCC4(C(=O)CO)O)C)O)F
4	Fluocinolone	NA	CC12CC(C3(C(C1CC(C2(C(=O)CO)O)O)CC(C4=CC(=O)C=CC43C)F)F)O
5	Clobetasol	NA	CC1CC2C3CCC4=CC(=O)C=CC4(C3(C(CC2(C1(C(=O)CCl)O)C)O)F)C
2831	OM-137	Aurora kinase inhibitor	CC1=C(SC(=N1)N)C(=O)NN=CC2=CC(=C(C=C2)O)OC
2832	QL-X-138	mTOR inhibitor	CC1=C(C=C(C=C1)N2C(=O)C=CC3=CN=C4C=CC(=CC4=C32)C5=CNN=C5)NC(=O)C=C
2833	GW-843682X	NA	COC1=C(C=C2C(=C1)N=CN2C3=CC(=C(S3)C(=O)N)OCC4=CC=CC=C4C(F)(F)F)OC
2834	Cycloheximide	NA	CC1CC(C(=O)C(C1)C(CC2CC(=O)NC(=O)C2)O)C
2835	Emetine	Protein synthesis inhibitor	CCC1CN2CCC3=CC(=C(C=C3C2CC1CC4C5=CC(=C(C=C5CCN4)OC)OC)OC)OC
2836	Gestrinone	Androgen receptor modulator	CCC12C=CC3=C4CCC(=O)C=C4CCC3C1CCC2(C#C)O
2837	MK-1775	NA	CC(C)(C1=NC(=CC=C1)N2C3=NC(=NC=C3C(=O)N2CC=C)NC4=CC=C(C=C4)N5CCN(CC5)C)O

^1^HDAC, histone deacetylase.

### Identification of candidate drugs by molecular docking and molecular dynamics simulation

**Figure 8:**
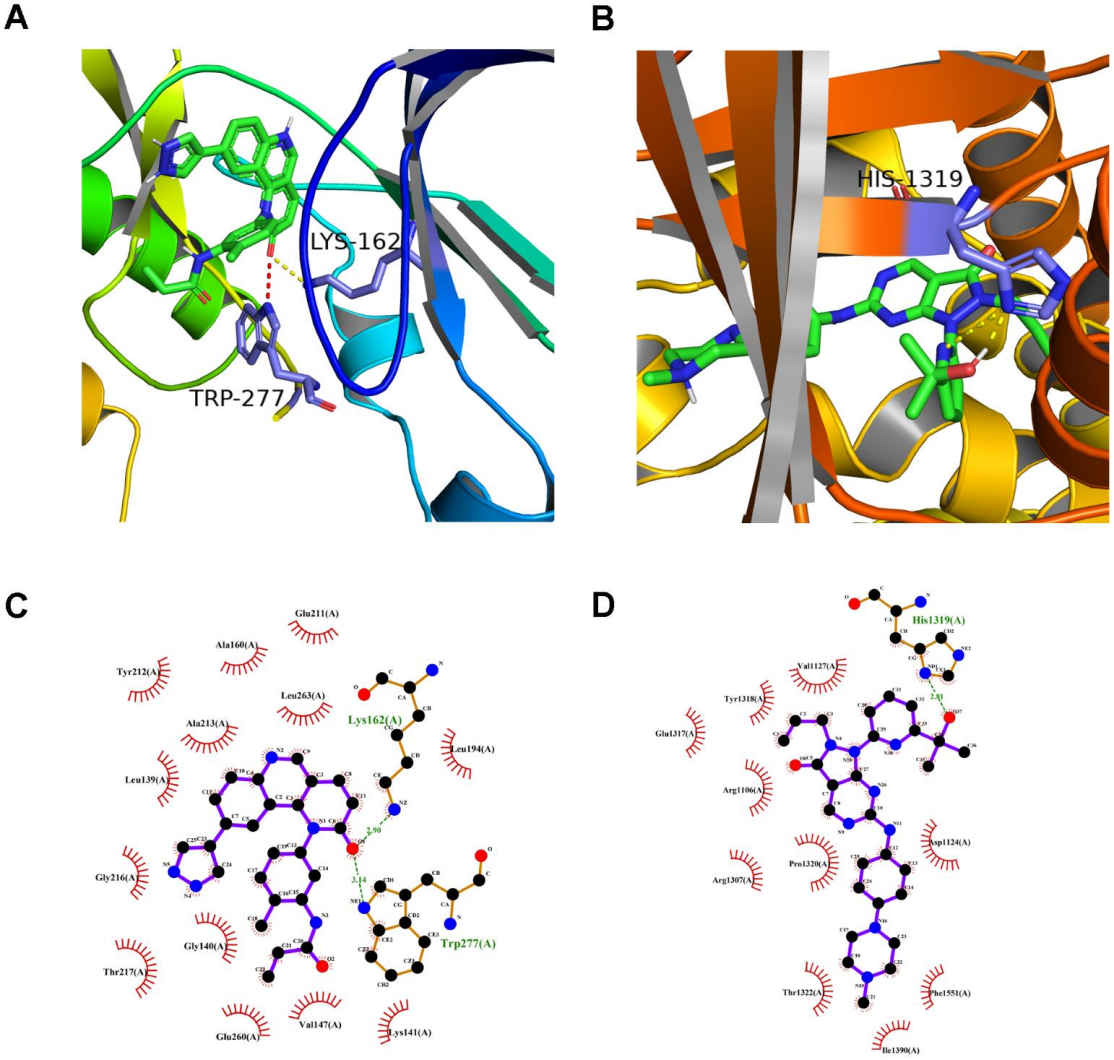
**The molecular docking mode of small compounds and proteins.** (**A**) The three-dimensional structure of interaction between QL-X-138 and AURKA. (**B**) MK-1775 and DUOX1. (**C**) The two-dimensional structure of interaction between QL-X-138 and AURKA. (**D**) MK-1775 and DUOX1. The yellow dotted line represents the hydrogen bond, while the red dotted line represents the pi-cation bond.

A preliminarily investigation was performed to verify whether there was direct targeting of the eight hub ferroptosis-related proteins of candidate drugs. The molecular docking results evaluated with binding energy have been presented in Table 2 (excluding the NOX4 and DUOX2 as unspecified protein structures). The small compound, QL-X-138, had the highest affinity but the lowest binding energy when targeted towards AURKA (-9.8 kcal/mol). Similarly, MK-1775 showed the highest affinity but the lowest binding energy when targeted towards DUOX1 (-9.3 kcal/mol). This analysis revealed the formation of two stable conformations with the lowest binding energies, among the 24 docking results. Next, the interactions between small compounds and amino acid residues were performed by Pymol and Ligplot. The ligand QL-X-138 formed a hydrogen bond with residue Lys162 and a pi-cation bond with Trp277 of protein AURKA (Figure 8A, 8C). While binding to DUOX1, MK-1775 formed a hydrogen bond with residue His1319 (Figure 8B, 8D). Two axes related to the two proteins in the ceRNA network were obtained (Figure 6D). The miRNAs and lncRNAs selected depend on the basis of their degree of connection might regulate AURKA and DUOX1 through ENSG00000248115-hsa-miR-335-AURKA and ENSG00000226828-hsa-miR-4265/hsa-miR-4296/hsa-miR-4322-DUOX1 axes at the transcriptome level, respectively.

**Table 2:** The predicted binding energy between candidate drugs and hub genes.

**Gene**	**PDB identifier**	**Test compound**	**Binding energy (kcal/mol)**
AURKA	1MUO	QL-X-138	-9.8
DUOX1	7D3F	MK-1775	-9.3
AURKA	1MUO	MK-1775	-9.1
AURKA	1MUO	GW-843682X	-9.1
DUOX1	7D3F	GW-843682X	-8.9
DUOX1	7D3F	QL-X-138	-8.6
VEGFA	1BJ1	MK-1775	-8.4
BNIP3	2KA2	QL-X-138	-8.4
BNIP3	2KA2	MK-1775	-8.0
HNF4A	1PZL	GW-843682X	-8.0
VEGFA	1BJ1	QL-X-138	-7.8
VEGFA	1BJ1	GW-843682X	-7.5
IL6	5FUC	GW-843682X	-7.3
BNIP3	2KA2	GW-843682X	-7.3
IL6	5FUC	MK-1775	-7.2
HNF4A	1PZL	OM-137	-7.0
IL6	5FUC	QL-X-138	-6.8
AURKA	1MUO	OM-137	-6.8
VEGFA	1BJ1	OM-137	-6.4
DUOX1	7D3F	OM-137	-6.3
IL6	5FUC	OM-137	-5.9
BNIP3	2KA2	OM-137	-5.9
HNF4A	1PZL	MK-1775	-5.4
HNF4A	1PZL	QL-X-138	-3.0

**Figure 9:**
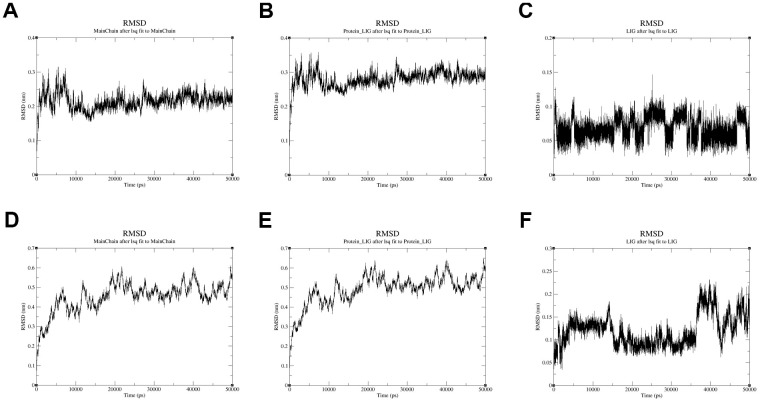
**The RMSD plot in the process of molecular dynamics simulation.** (**A**) The RMSD of protein AURKA. (**B**) The RMSD of protein-ligand QL-X-138 and AURKA system. (**C**) The RMSD of ligand QL-X-138. (**D**) The RMSD of protein DUOX1. (**E**) The RMSD of protein-ligand MK-1775 and DUOX1 system. (**F**) The RMSD of ligand MK-1775.

Moreover, the conformational stability of these two complexes was also verified by molecular dynamics simulation. The protein Calpha root-mean-square deviation (RMSD) data of QL-X-138 and AURKA, as well as MK-1775 and DUOX1, were obtained in terms of protein mainchains and protein-ligands. In comparison with the first frame in Figure 9A, 9D, the RMSD of the protein backbone of QL-X-138 and AURKA system became stable after 10 ns, with a deviation of 0.19. While the MK-1775 and DUOX1 system tended to become stable after 40 ns, with a deviation of 0.57. As for the protein-ligand RMSD, the system of QL-X-138 and AURKA, MK-1775 and DUOX1 both achieved equilibrium at 10 ns and 40 ns, with a deviation of 0.25 and 0.61, respectively (Figure 9B, 9E). Meanwhile, the RMSD of ligand QL-X-138 and MK-1775 was also stable at 5 ns and 36 ns as shown in Figure 9C, 9F, with a deviation of 0.09 and 0.14, respectively. Additionally, after reaching stability, the RMSD of the protein backbone, protein-ligand and ligand of the QL-X-138 and AURKA system maintained up to 45 ns. For all systems, the RMSD became stable after 40 ns. These data provided computational insight into the contribution of molecular dynamics simulation to the stable conformation of the systems of QL-X-138 and AURKA, MK-1775 and DUOX1.

## DISCUSSION

Since its discovery, ferroptosis has been associated with various human diseases, including cancer [24], neurological diseases [44], diabetes mellitus [45], and retinal degeneration [46] et al. However, to our knowledge, little attention has been paid to the involvement of ferroptosis in the pathogenesis of APA. In our study, we first identified a novel ceRNA regulatory network based on ferroptosis-related DEGs and DE lncRNAs. Furthermore, we explored candidate drugs that could target the eight hub genes for the APA treatment with molecular docking and molecular dynamics simulation. These findings revealed the pivotal genes involved in the regulation and therapy through ferroptosis signaling pathways. Although ferroptosis and immune infiltration have been implicated in pathogenesis of various tumors with abundant studies, little is known about the latent effect on APA [20, 33, 34]. Additionally, using transcriptomic and microarray sequencing analyses to identify the new biomarkers can improve APA diagnosis and treatment as they have been used in cancer [35].

We identified 89 ferroptosis-related DEGs associated with APA. The enrichment analysis of GO BP and KEGG pathways indicated that these genes mainly regulated cellular functions including responses to oxidative stress, reactive oxygen species metabolic process, superoxide metabolic process and HIF1 TFpathway. Morphological analysis revealed that ferroptosis occurs with elevated bilayer membrane density and decreased mitochondrial volume, resulting in cell membrane intact. Biochemical analyses have shown that glutathione depletion and lipid peroxide accumulation with reduced activity of GPX4 occurred in the process of ferroptosis [47]. Multiple signal molecules and metabolic pathways are involved in the occurrence and development of ferroptosis, such as HIF-1α, NADPH oxidase, GPX4, iron and lipid metabolism. Recently, a study in kidney injury demonstrated that ferroptosis induced by folic acid was decreased with FG-4592 pretreatment, an inhibitor of prolyl hydroxylase of HIF, through the Akt/GSK-3β/Nrf2 pathway [48]. Ferroptosis, enhanced by excess ROS production, injured renal tubules, and promoted diabetic nephropathy via HIF-1α/HO-1 pathway in db/db mice [49]. Among the eight hub genes, NOX4, a major ROS producer, catalyzes the conversion of oxygen to superoxide using NADPH [50]. Min et al. observed that NOX4 increased the astrocytic ferroptosis level and ferroptosis-dependent cytotoxicity through lipid peroxidation and impaired mitochondrial metabolism in Alzheimer's diseases [51].

The accumulation of ROS, a major inducer of ferroptosis, triggers autophagy when ferroptosis is induced by erastin [52]. Autophagy and ferroptosis are two types of programmed cell death that remain controversial regarding relationship, and ROS may become a ‘bridge’ connecting them. Damage to the cell membrane by ROS may be initiated from lipid peroxidation chain reaction which leads to the ferroptosis, autophagy, or apoptosis [39], indicating that ROS played a critical role in concatenating ferroptosis and other programmed cell deaths. AURKA is a cell cycle-regulating kinase that regulates the formation of mitotic spindle during chromosome segregation [53]. However, AURKA has been reported to be upregulated in several tumors. A recent study revealed that the suppression of AURKA caused cell death via GPX4, a well-known ferroptosis inhibitor [54]. The inhibition of GPX4 resulted in the accumulation of lipid-based ROS, thereby inducing ferroptosis [55], suggesting a strong link between AURKA and ferroptosis. In this study, we identified the lnc-RASL11B (ENSG00000248115)-miR-335-AURKA regulated network.

The proinflammatory cytokine IL6 participates in the IL6/Jak2/Stat3 pathway to regulate iron and maintain iron homeostasis [56]. However, high expression of IL6 disturbed iron homeostasis and subsequently induced ferroptosis by producing lipid peroxidation [57]. One transcription factor, HNF4A, identified as a ferroptosis suppressor mediates one category of genes called ferroptosis downregulated factors (FDF) by affecting the biosynthesis of glutathione in liver cancer. Additionally, disintegration of KAT2B preferentially prevented HNF4A from binding to FDF promoter and facilitated HIC1 binding to ferroptosis upregulated factors [58]. Besides ROS-related genes, pleiotropic cytokine, serine/threonine kinase and even gene regulated FDF are involved in ferroptosis.

In general, 26 DE lncRNAs constituting the ceRNA network were associated with ferroptosis. The top 10 lncRNAs regulate KEGG pathways, including renin-angiotensin system, steroid biosynthesis, calcium signaling pathway, primary immunodeficiency, chemokine signaling pathway, and natural killer cell mediated cytotoxicity. One study revealed that the overexpression of lncRNA KCNQ1OT1 mediated the upregulation of L-type Ca^2+^ channels, CACNA1C, by binding miR-384 in angiotensin-II induced atrial fibrillation [59]. In cultured HeLa cells, under low oxygen conditions, lncRNA MALAT1 was upregulated by the CaMKK/AMPK/HIF-1α axis [60]. Increasing evidence has demonstrated the effects of mutant genes that encoding calcium ion channels and pumps on the production of aldosterone. Elevated intracellular calcium concentration in zona glomerulosa cells of the adrenal cortex induces CYP11B2 overexpression and autonomous production of aldosterone [61]. Additionally, the overexpressed lncRNA MEG3 interacted with miR-125a-5p and suppressed its expression, whereas the downregulation of MEG3 increased the expression of FOXP3 via miR-125a-5p in the CD4^+^ T cells in immune thrombocytopenic purpura [62]. Silencing of lncRNA NEAT1 by siRNA repressed lipid uptake and the inflammatory response by targeting miR-342-3p in THP-1 macrophage, providing a strategy to treat atherosclerosis [63].

KCNJ5, which encodes the potassium channel GIRK4, is the most frequent mutation in APA. The prevalence of APA induced by KCNJ5 mutation was 34% in Europe [64], while 73% in Japan [65] and 70.7 % in China [66]. In patients with APA, KCNJ5 achieved a better prognosis than those with WT based on the PASO [16, 66]. In our results, NOX4 and DUOX2 showed significantly differentially expressed in KCNJ5 mutant samples compared to WT samples that the two ferroptosis-related genes may be involved in pathogenesis and prognosis. The inhibition of PIEZO1, a calcium channel, could decrease ferroptosis-related process such as ROS and lipid peroxidation in pulmonary endothelial cells, which is accompanied by calcium efflux [67], suggesting that calcium ion channel may impact the ferroptosis via calcium influx/efflux. ROS produced by NOX4 decreased the expression of Kv1.5 (a potassium channel) by activating P-Smad2/3 and P-ERK ½ [68], indicating that NOX4 may influence potassium channel by increasing ROS levels. However, there is little evidence regarding the effect of KCNJ5 mutation on the ferroptosis in APA, and NOX4 and DUOX2 may be novel targets for APA investigation and therapy.

Growing evidence demonstrated that various complicated diseases can scarcely be explained by one or a few genomic variations. Importantly, constructing a ceRNA network could help us understand the entire protein-coding genes dimension through the cross talk of non-coding RNAs. The interaction between non-coding RNAs based on the ceRNA can regulate the post-transcription levels of genes. According to the ceRNA hypothesis, miRNA response elements act as letters of “RNA language” to communicate with mRNAs and lncRNAs to regulate their expression. RNAs crosstalk is efficient when they share numerous miRNA response elements [43]. In this study, we identified the key DEGs and DE lncRNAs associated with ferroptosis in APA. The miRWalk 2.0 database predicted 132 miRNAs that targeted four hub genes and regulated 26 DE lncRNAs to construct the ceRNA network. In addition, well-defined lncRNAs can be functionalized by ceRNA involved in diverse mechanisms to balance the relationship between mRNAs and shared miRNAs. KCNQ1OT1 functioned as a ceRNA and stimulated the expression of HDAC3 by competitively binding to miR-452-3p, thereby repressing ABCA1 expression and lipid accumulation in macrophage [69]. The lncRNA LINC00336 acted as a sponge to inhibit miR-6852 and regulated ferroptosis by cystathionine-β-synthase. LINC00336 inhibited ferroptosis by binding to the RNA-binding protein ELAVL1 [70], implying that lncRNAs may play multiple roles in crosstalk in the ceRNA network.

In considering new insights regarding APA treatment, we used CMAP to screen for possible candidate drugs; it was developed by CMAP team of the Broad community, and the data are publicly available [71]. In the CMAP database, drug signatures fit over one million gene expression profiles obtained from approximately 50 thousand unique perturbagens and are identified by computing algorithms, thereby making the discovery of new drugs or identification of novel uses for existing drugs more efficient. Similar to the expression change between parbendazole and osteogenic hMSCs, Andrea et al. identified a novel bone anabolic compound that caused osteogenic differentiation by altering the activity between BMP-2 and cytoskeleton [72]. CMAP analysis revealed different enrichment for CX3CR^+^ and CX3CR^-^ gene sets of single lung interstitial macrophage subpopulations [73]. We uploaded 44 upregulated genes and 45 downregulated genes in APA to query CMAP, and the differential expression profile was fit to the drugs identified using the Kolmogorov-Smirnov statistical test. Fludrocortisone, a synthetic mineralocorticoid, is involved in the top ranked drugs that leads to modest hyperaldosteronism [74]. The utilization of this category of drugs may cause clinical characteristics similar to those of APA. Therefore, the bottom ranked drugs were selected to determine the targeted treatment of APA through ferroptosis, *in silico*.

To avoid repetitive experiments and increase the research efficiency, bioinformatics technology is a highly efficient approach for predicting novel therapeutic drugs for diseases in a low-cost manner. In our study, QL-X-138 and MK-1775 bound to the AURKA and DUOX1, respectively, with the lowest binding energies in molecular docking. As MK-1775 is known as a WEE1 inhibitor, we analyzed its binding energy as a positive control to measure the affinity of docking results. The binding energies were -9.8 kcal/mol for the QL-X-138 and AURKA complex, -9.3 kcal/mol for the MK-1775 and DUOX1 complex and close to -10.3 kcal/mol for the MK-1775 and WEE1 complex. This indicated that QL-X-138 and MK-1775 had good binding activities with their targeted proteins and could be candidate drugs for further experimental verification. In addition, according to the molecular dynamics simulation, the RMSD stability is indispensable to deduce a good binding affinity [75]. The complexes of QL-X-138 and AURKA, MK-1775 and DUOX1 revealed stable RMSD in the simulated results. Molecular dynamics simulation is frequently used to predict how biomolecules, especially proteins, will respond at the atomic level when perturbed [76]. Along with its more popular application in recent years, molecular dynamics simulation has helped to design new drugs such as opioid analgesics with reduced side effects [77] and the development of existing drugs as selective agonists compared to the traditional opioid fentanyl [78]. Drug resistance is a barrier to chemotherapy in tumor related therapies. The ceRNA network of drug-resistant constructed in non-small cell lung cancer offered a novel approach to solving this problem [79]. Given that the current drugs have severe side effect in treatment of APA, ceRNA construction could help us to find novel candidate drugs with bioinformatic analyses that have been used in cancers. In this study, AURKA and DUOX1 acted as drug targets and the regulatory axis of ceRNA could be a promising therapeutic strategy for further studies.

In the present study, we provided insights into the pathogenesis of APA at the transcriptome level, explored the relationship between ferroptosis and APA, and established a methodological reference for further confirmation of experiments. Nevertheless, the sample set size for analysis was relatively small, and a large set of APA samples would be necessary to conduct *in vivo* and *in vitro* experiments to verify our findings.

## MATERIALS AND METHODS

### Data collection

We obtained microarray data associated with APA and AAG tissue from GEO database. The datasets for mRNA expression profiling were screened as follows: (1) Homo sapiens datasets; (2) datasets contain more than 10 APA samples; (3) number of gene was more than 10000; (4) datasets extra contain lncRNA or KCNJ5 mutation samples expression profiles. At last, datasets GSE64957 and GSE101894 including mRNA and lncRNA expression profiling were collected to analysis DEGs and DE lncRNAs. The dataset GSE64957 contained transcriptome profile of zona glomerulosa (ZG), zona fasciculata (ZF) and APA, and ZG was chosen for comparison as the AAG, since the ZG was known as the zone of aldosterone synthesis and secretion in adrenal gland [17]. Datasets GSE156931 and GSE64957 including KCNJ5 mutation and wild type (WT) samples were collected to analysis hub genes expression between KCNJ5 mutation and WT.

We obtained 263 ferroptosis-related genes from FerrDb [80] which was an integrated and up-to-date database involving ferroptosis-related genes, molecules and diseases.

### Data normalization and identification of ferroptosis-related DEGs

Differential expression analysis of GSE64957 was performed on the GEO2R online tool; the original files of GSE101894 and GSE156931 were downloaded from GEO and normalized by Sangerbox tools (http://www.sangerbox.com/tool) as described before [81]. The expression profiles of APA and AAG samples were compared to identified DEGs and DE lncRNAs. The screening criteria were | log2 (fold-change) | > 0.5 and adjusted *P* < 0.05. We selected the most significant ones when the DEGs or DE lncRNAs were duplicated. A venn diagram of DEGs and ferroptosis-related genes was drawn by Venny 2.1 (http://bioinfogp.cnb.csic.es/tools/venny/index.html).

### Volcano plot and heatmap plot analysis

To better visualize the ferroptosis-related DEGs, the volcano plot and heatmap were created by Excel and Helm software, respectively.

### Enrichment analysis

Metascape (http://metascape.org) is a reliable platform for gene list enrichment analysis [36]. Based on the function, we used the Custom Expression module to verify the enriched and related neighbor pathway of ferroptosis-related DEGs. The chord plot was drawn by Sangerbox tools.

GSEA software was used to assess the correlation of the lncRNAs in a predefined set of RNA table to other genes in regulating Kyoto Encyclopedia of Genes and Genomes (KEGG) pathways. Statistical significance was set at *P* < 0.01 and false discovery rate *q* < 0.25.

### PPI network construction

The ferroptosis-related DEGs in APA were uploaded to Metascape tool with Custom Expression module to obtain the PPI network. MCODE algorithm was then applied to this network to identify neighborhoods where proteins were densely connected. GO enrichment analysis was applied to each MCODE network to assign “meanings” to the network component. We can identify the physical and combined cores with this algorithm.

### Screening for hub ferroptosis-related DEGs

The normalized data was analyzed by WGCNA package in R-Studio 3.6.1 software [41]. The application of WGCNA could group genes into modules based on their co-expression patterns. Thus, it can establish a connection between the characteristics of the samples (such as disease status or treatment) and changed expression levels of genes. This can help researchers identify potential disease-related genes or pathways. Also, it offered a systemic insight into the regulatory network and signaling pathway which may be related to the clinical traits. So clinical traits-related modules were constructed to identify the hub genes with WGCNA [82]. Topological overlap matrix (TOM) was created by transforming adjacency matrix, so that constructed network accorded with the real biological network state. Based on the TOM dissimilarity, genes were divided into several modules. Then the scale- free network was constructed to identify the gene co-expression modules with similar expression patterns. And the modules were defined by cutting the clustering tree into branches with a dynamic tree algorithm. Different colors were given for better visualization [83]. All modules were assembled by module eigengenes, and the principal component of the modules that were figured as a synthetic gene stand for the gene expression profiles [83]. Here, we set the soft-thresholding power as 8, and minimal module size as 50. Correlation between clinical traits and module eigengenes were calculated [82]. The hub ferroptosis-related DEGs were verified from the most correlative modules eigengenes and ferroptosis-related DEGs.

### Expression of hub ferroptosis-related DEGs in mutant samples

To compare the expression level of hub ferroptosis-related DEGs in KCNJ5 mutation and WT APA samples, box-plots were drawn with datasets of GSE156931 and GSE64957 by Sangerbox tools. The datasets GSE156931 and GSE64957 were integrated with R package Siliconmerging and corrected of batch effect with ComBat in R package SVA to compare the expression of eight hub genes [81]. The principal component analysis (PCA) was used with R package Stats.

### Correlation analysis between hub ferroptosis-related DEGs and DE lncRNAs

The relationship between hub ferroptosis-related DEGs and DE lncRNAs was assessed using Pearson correlation. The association was considered significant if the correlation coefficient | *R^2^* | > 0.5 at *P* < 0.001. The correlative plot was drawn with the top 10 most correlative and highest connecting degree DE lncRNAs and hub ferroptosis-related DEGs.

### Prediction of target miRNAs

miRDB, PITA, RNA22, miRWalk, miRanda, RNAhybrid, and Targetscan databases in miRWalk 2.0 online tools [84] were used to predict the target miRNA of hub ferroptosis-related DEGs. We selected miRNAs that were found in at least five databases. LncBase online tool [85] was used to predict miRNAs of DE lncRNAs to identify the lncRNAs with the highest degree of connection.

### Construction of ceRNA network

The intersections of predicted miRNAs by hub ferroptosis-related DEGs and DE lncRNAs were selected to construct lncRNA-miRNA-mRNA ceRNA network. Based on the ceRNA network theory [43], the lncRNA positively correlated to the mRNA would be selected. The lncLocator was used as a subcellular localization predictor to further screen lncRNA [86]. Cytoscape software was used to depict network plot and found the mRNAs and lncRNAs with the highest degree of connection.

### CMAP analysis for candidate drugs

CMAP (https://clue.io/) is a database that explores the relationships between cell physiology, diseases and therapies, and it contains over one million transcriptional expression profiles perturbed by small molecular compounds [71]. Based on this database, we can find the positive and negative drugs associated with diseases which contains a similar expression profile. We uploaded the ferroptosis-related DEGs consisted with 44 upregulated genes and 45 downregulated genes. The bottom ranked drugs resulted in opposite expression profiles compared to those by diseases, and might have a therapeutic effect.

### Molecular docking

The protein 3D structure of eight hub ferroptosis-related DEGs were downloaded from protein data bank (https://www.rcsb.org/). And the structures of drugs targeted to these proteins were downloaded from ZINC (http://zinc.docking.org/). We first removed ligand and water macromolecule, and added hydrogen atoms with Pymol 2.5. The proteins were set to rigid and determined as root. Second, we set twisted key to flexible saved as pdbqt file format by AutoDock Tools 1.5.6. At last, Autodock Vina 1.1.2 was used to computing the molecular docking. The conformations with lowest binding energy were selected for further analysis of their interactions and binding patterns through Pymol 2.5 and Ligplot 2.2.

### Molecular dynamics simulation

Following the molecular docking, we tested the stability of the drugs binding to the proteins with lowest binding energy. The force field for ligand and the partial charges were obtained by AMBER 18 with the antechamber module. The standard AMBER force field (ff99SB) [87] for protein and the general AMBER force field (GAFF) [88] for ligand were used in the molecular dynamics simulation by GROMACS 5.1.2 software. Each complex system was immersed in a rectangular box of TIP3P with water molecules, which expanded 12 Å away from any solute atoms. Next, we added proper numbers of Na^+^ or Cl^-^ to neutralize the complex systems. After minimizing energy, each complex system was heated in from 0 to 300K within 300 ps. Under stationary temperature of 300K for complex, a 50ns molecular dynamics simulation with a step of 2 fs was performed. In the process of sample, the coordinates were saved every 2 ps for subsequently free energy calculation and free energy decomposition analysis.

### Statistical analysis

Student's t-test was used to compare the gene expression of KCNJ5 mutation and WT APA samples. Pearson correlation was used to analysis the relationship between mRNAs and lncRNAs. These were performed on the Sangerbox tools.

### Data availability statement

The raw data supporting the conclusions of this article will be made available by the authors, without undue reservation. Some codes are available on GitHub (https://github.com/aldosteronelab/WGCNA).

## Supplementary Material

Supplementary Figure 1
