# Supplementary material for: Identification of a novel competing endogenous RNA network and candidate drugs associated with ferroptosis in aldosterone-producing adenomas
Source: Aging (Albany NY). 2023 Sep 13;15(17):9193–216. doi: 10.18632/aging.205028 (PMC10522391; doi:10.18632/aging.205028)
Supplement: Supplementary Figure 1 [file aging-15-205028-s001.pdf]

## SUPPLEMENTARY FIGURES

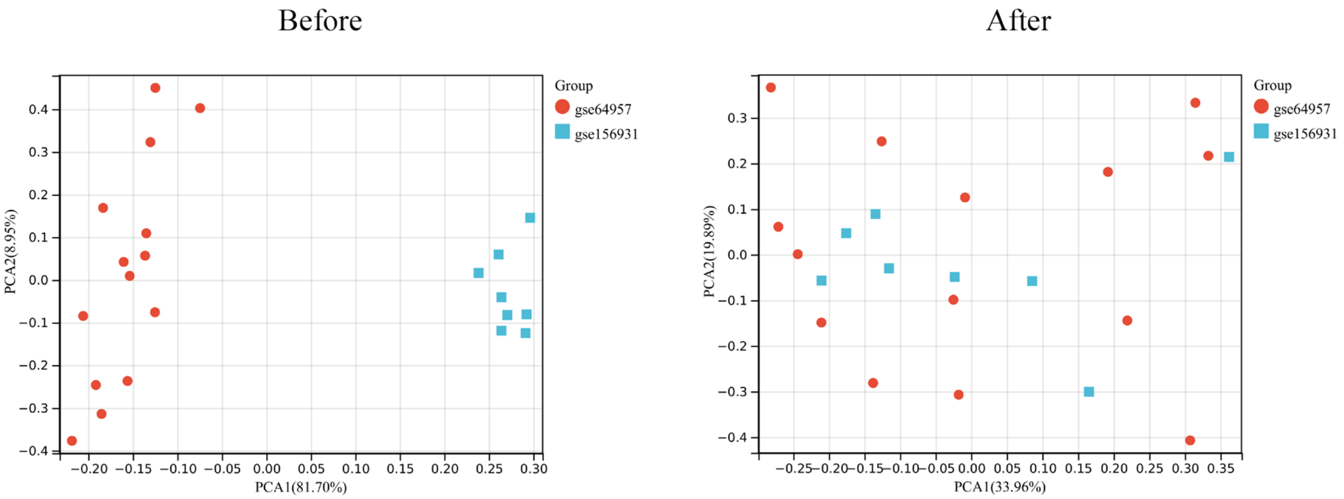


**Supplementary Figure 1. The PCA diagram before and after batch correction.** The datasets GSE156931 and GSE64957 were integrated and corrected of batch effect.
